# Supplementary material for: Noncoding RNAs: Master Regulator of Fibroblast to Myofibroblast Transition in Fibrosis
Source: Int J Mol Sci. 2023 Jan 16;24(2):1801. doi: 10.3390/ijms24021801 (PMC9861037; doi:10.3390/ijms24021801)
Supplement: Supplementary file 1 [file ijms-24-01801-s001.zip › Table S1.pdf]

Table S1. the potential ncRNA biomarker for FMT.

Noncoding RNA	effects	expression	target	disease	reference
MicroRNA					
miR-369-5p	inhibit	Down-regulation	Over-expression of miR-369-5p decreased the expression of COL1A1 and α-SMA	Myocardial fibrosis	[117]
miR-199a（5p/3p）	promote/inhibit	Up-regulation	① Activate PI3K/AKT/MTOR signaling pathway<br>② Target caveolin-1	Intimal cord fibrosis/skeletal muscle fibrosis/pulmonary fibrosis	[118-122]
miR-29（a/b/c）	inhibit	Up-regulation / Down-regulation	① Target VEGF-A<br>② Target Smad7，α-SMA<br>③ Target TPM1	Corneal fibrosis/high myopia/myocardial fibrosis/pulmonary fibrosis/renal interstitial fibrosis/oral submucosal fibrosis	[26, 36, 38, 74, 102, 116, 123, 124]
miR-129-3p	promote	Down-regulation	Target Smad3	Myocardial fibrosis	[125]
miR-23/27b-3p	promote	Up-regulation	Target TGFβR3，activate TGF-β/Smad signaling pathway	Atrial fibrillation due to myocardial fibrosis	[49]
miR-21	promote	Up-regulation	① Target PDCD4<br>② Affect ADAMTS-1/TGF-β signall pathway，degrade ECM<br>③ Target TGF-RIII<br>④ Target Spry<br>⑤ Target PTEN， inhibit p-AKT/P-MTOR<br>⑥ Target smad7<br>⑦ Target jagged1<br>⑧ Target TGF-β2	Systemic sclerosis/revascularization/myocardial fibrosis/scar/breast cancer	[68, 76, 83, 87, 95, 96, 111, 115, 126-136]
miR-146a	inhibit	Up-regulation / Down-regulation	① Target Smad4<br>② Target CXCR4<br>③ Target C0-C1f	Diabetic cardiomyopathy/myocardial fibrosis	[63, 137]
miR-208a	promote	Up-regulation	Target DYRK2	myocardial fibrosis	[138]
miR-let-7b/b/p	inhibit	Down-regulation / up-regulation	① Target IL-8<br>② Target TGFBR1	Breast cancer/pulmonary fibrosis	[24, 42, 139, 140]
miR-101a	inhibit	Down-regulation	Target TGFβR1	myocardial fibrosis	[40]
miR-7a/b	inhibit	Down-regulation	Target Smad2	myocardial fibrosis / pulmonary fibrosis	[141]
miR-214	inhibit /	Down-	① Target TGF-β1	myocardial fibrosis	[142-144]

	promote	regulation	② Target TIMP1<br>③ Target NLRC5		
miR-127-3p	inhibit	Down-regulation	Regulate the expression of MAPK4、COL Ⅰ/Ⅱ、α-SMA	Airway scar	[88, 145]
miR-34a	inhibit	Down-regulation	① Target FLIPS<br>② Target SIRT1<br>③ Target C-ski	Liver fibrosis/pulmonary fibrosis/myocardial fibrosis	[146-148]
miR-30c/d	inhibit	Down-regulation	① Target TGFβR II<br>② Target α-ZAP<br>③ Target JAG1	myocardial fibrosis / pulmonary fibrosis	[46, 110, 149]
miR-145	promote	Up-regulation	① Target KLF4<br>② modulate various components of TGF-β signaling pathway	Pulmonary interstitial fibrosis/myocardial fibrosis	[150-158]
miR-126	inhibit	Down-regulation	① target DKK<br>② target PIK3R2	Rheumatoid arthritis	[75, 159]
miR-335-3p	inhibit	Down-regulation	target smad2/3	Hereditary gingival fibromatosis	[56]
miR-22	inhibit	Down-regulation	① Inhibition of ERK1/2 signaling pathway and negative regulation of CTGF expression<br>② Target CAV3	myocardial fibrosis / pulmonary fibrosis	[92, 160]
miR-10a/b	inhibit / promote	Down-regulation	① Target TGFβ1<br>② TWIST can promote the expression of Mir-10b and promote the progression of fibrosis	oral fibrosis/myocardial fibrosis	[161, 162]
miR-155	promote	Up-regulation	① Promote the expression of COL Ⅰ/Ⅱ、IL-6、IL-1β、TNF-α<br>② Target TP53INPI<br>③ Target AT(1)R，ERK1/2 activation was inhibited	Myocardial fibrosis/intimal vascular fibrosis	[91, 163-166]
miR-200a/b/c	inhibit	Down-regulation	① Inhibit the expression of SIRT1,α-SMA<br>② Target ZEB2<br>③ Target ZEB1	Myocardial fibrosis/oral submucosal fibrosis/buccal mucosa fibrosis	[113, 114, 167]
miR-195	inhibit / promote	Up-regulation / Down-regulation	Target Check1	Myocardial fibrosis	[168]
miR-125b	promote	Up-regulation	① Target Sirtuin7<br>② Target SFRP5<br>③ Modulate RhoA signaling pathways	Liver fibrosis/myocardial fibrosis/aging skin damage	[169-172]
miR-27a-	inhibit	Down-	Inhibit the expression of α-SMA、	myocardial fibrosis	[52, 64, 103]

3p		regulation	smad2、smad4		
miR-140	promote / inhibit	Up-regulation / Down-regulation	Inhibitor TGF-β1signaling pathway	myocardial fibrosis	[173]
miR-375	inhibit	Down-regulation	Target P38	pulmonary fibrosis	[174]
miR-327	promote	Down-regulation	-	myocardial fibrosis	[175]
miR-133a	inhibit	Down-regulation	① Target Furin<br>② Target TGFBR1 , inhibit the expression of CTGF、COL Ⅰ a1	Thoracic aortic aneurysm / pulmonary fibrosis	[176, 177]
miR-144	inhibit	Down-regulation	Target CREB	myocardial fibrosis	[178]
miR-210-5p	inhibit	Up-regulation	Target STAT5A	Fibrosis of skin	[107]
miR-192	promote	Up-regulation	Target SIP1（Smad interacting protein）	Hypertrophy scar fibrosis	[179]
miR-574-5p	promote	Up-regulation	Target ARID3A	myocardial fibrosis	[180]
miR-127-3p	promote	Up-regulation	-	Myofibroblast senescence phenotype	[88]
miR-152-3p	inhibit	Down-regulation	Inhibit wnt1/ β -catenin signaling pathway	myocardial fibrosis	[181]
miR-370	inhibit	Up-regulation	Inhibit the expression of TGF-R2/smad3	myocardial fibrosis	[47]
miR-130	inhibit / promote	Down-regulation / up-regulation	① Target TGFβR1<br>② Target peroxidase	myocardial fibrosis	[41, 182]
miR-218	promote	Down-regulation	Target Cezanne （ deubiquitinating enzymes）	Gingival fibroblasts	[183]
miR-26	inhibit	Down-regulation	① Modulate TGF- β signaling pathway<br>② Target Keap1	Pulmonary artery hypertrophy/myocardial fibrosis	[184, 185]
miR-33a-3p	inhibit	Up-regulation	Target DKK-1	Systemic sclerosis	[100]
miR-338-3p	inhibit	Down-regulation	①Target FGFR2<br>②Target PTEN	myocardial fibrosis	[77, 90]
miR-154	promote	Up-regulation	Target DKK2	myocardial fibrosis	[101]
miR-150	inhibit	Up-regulation	Modulate the expression of c-myb	myocardial fibrosis	[186, 187]
miR-541-5p	inhibit	Down-regulation	Modulate the expression of PDE1A	pulmonary fibrosis	[188]
miR-1246	promote	Up-regulation	Might target COL Ⅰ	Oral submucosal fibrosis	[189]
miR-216a	promote	Up-regulation	target PTEN	Renal fibrosis	[190]

miR-16-5p	inhibit	Down-regulation	Inhibit NOTCH signaling pathway	Systemic sclerosis	[109]
miR-497	promote	Up-regulation	-	Oral submucosal fibrosis	[191]
miR-433	promote	Up-regulation	① Target AZIN1，repress TGF-β1；<br>② Target JNK1	myocardial fibrosis	[23]
miR-9-5p	inhibit	Down-regulation	Target TGFβR2	Fibrosis of the human dermis	[43, 48, 192]
miR-375	inhibit	Down-regulation	Target FOXF1	Fibrosis of mucosa	[193]
miR-122	inhibit	Down-regulation	Target FN1	Liver fibrosis	[194]
miR-503	inhibit	Down-regulation	Target VEGFA、FGFR1	pulmonary fibrosis	[78]
miR-449	inhibit	Down-regulation	Target PLOD1	Skin scar	[195]
miR-320	promote	Up-regulation	Activate PI3CA/AKT/MTOR signaling pathway	myocardial fibrosis	[196]
miR-32-5p	promote	Up-regulation	Target DUSP1	myocardial fibrosis	[89]
miR-424	promote	Up-regulation	Target Slit2	pulmonary fibrosis	[197]
miR-877-3p	inhibit	Down-regulation	Target Smad7	myocardial fibrosis	[69]
miR-192-5p	inhibit	Up-regulation	Target IL-17RA , modulate Smad signaling pathway	Fibrosis of scar	[198]
Circular RNA					
circHIPK3	promote	Up-regulation	① Competitive binding with mir-338-3p promotes the expression of SOX4 and COL1A1<br>② Competitive binding with mir-30-3p promotes the expression of FOXK2 and promotes glycolysis<br>③ Competitive binding with miR-152-3p promotes the expression of TGF-β2	pulmonary fibrosis / myocardial fibrosis	[22, 31, 32, 199-202]
Mmu-circ-0005019/circ-0099734	inhibit	Down-regulation	Competitive binding with miR-499-5p promotes the expression of kcdn1/kcdn3/scn5a/kcnn3	myocardial fibrosis	[203]
circAMD1	inhibit	Down-regulation	Competitive binding with miR-27a-3p inhibits functional and phenotypic changes in p63 mutant human dermal fibroblasts	p63 mutant human dermal fibroblasts	[53]
circNLgn	promote	Up-regulation	NLgn173 binds to laminB1 and localizes it to the nucleus. NLgn173 binds to the ING4 and C8orf44GSK3 promoters,	Myocardial remodeling	[204]

			resulting in abnormal deposition of collagen and class switching of fibroblasts.		
circ0044226	promote	Up-regulation	Competitive binding with miR-7 promotes sp1 expression	pulmonary fibrosis	[205]
circCUL2	promote	Up-regulation	Competitive binding with miR-203a-3p activates MYD88/NF-κb pathway to transform pancreatic fibroblasts into cancer-associated fibroblasts, and secretes IL-6 to promote PDAC progression.	Double adenocarcinoma of pancreas	[206]
circfgfr2	promote	Up-regulation	Competitive binding with miR-133 promotes the expression of MAP3K20/JNK/MAPK/KLF4, while KLF4 can bind to the circFgfr2 promoter to promote the expression of circFgfr2	Muscle regeneration	[97]
circ-010567	promote	Up-regulation	competitively binds with miR-141 and promotes the expression of TGF-β	myocardial fibrosis	[207]
circ-SCAR	inhibit	Down-regulation	Direct binding to ATP5B inhibits mitochondrial mROS output and class transformation in fibroblasts	Non-alcoholic steatohepatitis	[208]
circHECTD1	promote	Up-regulation	Competitive binding with miR-142-3p promotes the expression of HMGB1, and then activates TGF-β/Smad pathway	Hypertrophic scar	[209]
circ-EP400	promote	Up-regulation	deriving from M2 macrophages exosomes competitively bind to miR-15b-5p and promote the expression of FGF1/7/9	Tendon injuries	[94]
circ-HNRNPH1	inhibit	Up-regulation	Competitive binding with miR-216-5p promotes smad7 expression and then promote TGF-β1 degradation	Myocardial fibrosis occurs after ischemia	[70]
circ-0047339	promote	Up-regulation	Competitive binding with miR-4691-5p promotes the expression of TSP-1(an endogenous activator of TGF-β)	Urethral scar fibrosis	[210]
Mmu-circ-37492/has-circ-0012138	inhibit	Up-regulation	Competitive binding with miR-7682-3p promotes the expression of FGB	Renal fibrosis	[211]
lncRNA					
TUG1	promote	Up-regulation	Activate TGF-β/Smad signaling pathway	Human periodontal membrane fibroblasts	[39]
DNM3OS	promote	Up-regulation	① Activate TGF-β/Smad signaling pathway<br>② Competitive binding of miR-199a-	myocardial fibrosis / pulmonary fibrosis	[212]

			5p activates TGF-β signaling pathway		
Fendrr	inhibit	Down-regulation	Binding to DNMT3B (DNA methyltransferase) promotes methylation of RASSF1A (RAS-associated region family 1A gene)	myocardial fibrosis	[93]
MEG3	inhibit	Down-regulation	-	myocardial fibrosis	[213]
n341773	inhibit	Down-regulation	Inhibit PI3K/AKT/mTOR signaling pathway	pulmonary fibrosis	[80]
MIR155HG	promote	Up-regulation	Competitive binding of miR-627 promotes the expression of HMGB1	pulmonary fibrosis	[214]
GAS5	inhibit	Down-regulation	① Might competitively binding of miR-21<br>② inhibits the expression of p-Smad and α-SMA<br>③ binds smad3, promotes the binding of smad3 to PPM1A (protein phosphatase 1A), and dephosphorylates smad3	myocardial fibrosis / Oral submucosal fibrosis	[54, 58-60, 82, 84, 215-218]
ASLNCS5088	promote	Up-regulation	Derived from M2 macrophage exosomes, it competitively binds miR-200c-3p and promotes GLS (glutaminase) and α-SMA expression	Hypertrophic scar	[219]
HOTAIR	promote	Up-regulation	Through H3K27me3-mediated activation of notch signaling pathway to induce FMT/ directed EZH2 (histone methyltransferase) can induce specific target gene H3K27me3, inhibit miR-34a expression, and activate NOTCH signaling pathway	Systemic sclerosis	[112, 220]
CTD-2528L19.6	inhibit	Early stage: Up-regulation Advanced stage: Down-regulation	Down-regulate fibrosis-related genes LRRC8C, DDIT4, THBS1, S100A8 and TLR7	Idiopathic pulmonary fibrosis	[221]
ZFAS1	promote	Up-regulation	Competitive binding of miR-150-5p promotes SLC38A1 expression	pulmonary fibrosis	[222]
Cfast （AK048087）	promote	Up-regulation	Binding to COTL1, competitively inhibits COTL1 interaction with TRAP1 (transforming factor receptor-β-associated protein 1), which enhances smad2/4 complex formation and TGF-β signaling	myocardial fibrosis	[223]

PFL(NONMMUT022555)	promote	Up-regulation	Competitive binding of let-7d promotes the expression of PTAFR (platelet activating factor)	myocardial fibrosis	[224]
Safe（AK 137033）	promote	Up-regulation	The RNA binding protein can be combined with the adjacent gene sfrp2 mRNA and lncRNA safe complex	Myocardial injury in rats	[105]
PFAL（MONMMUT021928）	promote	Up-regulation	Competitive binding of miR-18a promotes the expression of CTGF	pulmonary fibrosis	[81]
Rian	inhibit	Down-regulation	Over-expression of Rian or downregulation of Miat could reduce the expression of a-SMA, COLA1 and smad2/3	Renal fibrosis	[225]
Miat	promote	Up-regulation			
TUG1	promote	Up-regulation	Competitively bind of miR-29c	myocardial fibrosis	[39]
H19	promote	Up-regulation	Derived from M2 macrophages, it competitively binds let-7a/ competitively binds miR-29b to promote cola1 expression	pulmonary fibrosis / Oral submucosal fibrosis	[37, 226]
NEAT1	promote	Up-regulation	Competitive binding of miR-760 promotes the expression of TPM1	Oral submucosal fibrosis	[227]
IAPF	promote	Up-regulation	H3K27ac activates the IAPF promoter, ATF3 binds to the IAPF promoter, IAPF transcription is enhanced, and it forms RNA-protein complex with ELAVL1/HUR, inhibits the fusion of autophagosomes and lysosomes, and controls the stability of EZH2, STAT1 and FOXK1 to inhibit autophagy	pulmonary fibrosis	[228]
LOC103691771	promote	Up-regulation	Activate TGF-β-Smad2/3 signaling pathway	silicosis	[229]
SNHG7	promote	Up-regulation	Competitive binding of miR-34-5p promotes ROCK1 expression	Myocardial remodeling	[230]
HOTTIP	promote	Up-regulation	Promote the expression of a-SMA、COLI、IL-6、TNF-a	myocardial fibrosis	[231]
SNHG1	promote	Up-regulation	Competitive binding of miR-326 promoted the expression of SP1	pulmonary fibrosis	[232]
LINC01518	promote	Up-regulation	Competitively binds of miR-216b-5p	glaucoma	[233]
CRNDE	inhibit	Up-regulation	Smad3 promotes CRNDE, while CRNDE inhibits smad3 transcriptional activation of target genes	Diabetic myocarditis	[55]
LINC00312	promote	Up-regulation	The expression of a-SMA and p-Smad was promoted by YBX1	Oral submucosal fibrosis	[234]
SNHG20	promote	Up-regulation	Competitive binding of miR-490-3p promotes the expression of TGFBR1	silicosis	[45]

LINC00963	inhibit	Down-regulation	Competitively binds miR-143-3p	Corneal fibrosis	[235]
